# La coagulation intravasculaire disséminée: intérêt du score de la société internationale sur la thrombose et l´hémostase

**DOI:** 10.11604/pamj.2020.36.154.20368

**Published:** 2020-07-06

**Authors:** Sawsen Bouzidi, Mayssa Daiki, Amen Allah Nasr, Kaouther Nsiri, Samy Layouni, Zied Hajjej, Najiba Fekih-Mrissa, Mustapha Ferjani, Brahim Nsiri

**Affiliations:** 1 Laboratoire d´Hématologie, Hôpital Militaire Principal d´Instruction de Tunis, Tunis, Tunisie,; 2 Service d´Anesthésie Réanimation, Hôpital Militaire Principal d´Instruction de Tunis, Tunis, Tunisie,; 3 Unité de Recherche de Biologie Moléculaire (UR17DN06), Laboratoire d´Hématologie, Hôpital Militaire Principal d´Instruction de Tunis, Tunis, Tunisie

**Keywords:** Coagulation intravasculaire disséminée, score, société internationale sur la thrombose et l’hémostase, Disseminated intravascular coagulation, score, international society on thrombosis and haemostasis

## Abstract

La coagulation intravasculaire disséminée (CIVD) est une cause de mortalité redoutable en milieu de réanimation. L´utilisation du système de score de la société internationale sur la thrombose et l´hémostase (ISTH) permet de faciliter le diagnostic précoce de la CIVD. Nous présentons trois observations cliniques de CIVD d´étiologies différentes: un adénocarcinome prostatique, un choc septique et un hématome rétro-placentaire. Les tests d´hémostase nécessaires au calcul du score de la Société Internationale sur la Thrombose et l´Hémostase (ISTH) (numération plaquettaire, taux de prothrombine, fibrinogène et D-dimères) ont été régulièrement réalisés. D´autres tests complémentaires (recherche de complexes solubles, test de lyse des euglobulines, dosage des taux d´antithrombine, de protéine C activée et du facteur V) ont été également réalisés. L´utilisation du score ISTH permet de faciliter le diagnostic précoce de la CIVD.

## Abstract (en)

Disseminated intravascular coagulation (DIC) is a life-threatening event during resuscitation. The International Society on Thrombosis and Haemostasis (ISTH) diagnostic scoring system enables early diagnosis of DIC. We here report three clinical cases of DIC characterized by several etiologies: prostatic adenocarcinoma, septic shock and retroplacental hematoma. The tests of hemostasis needed to calculate international society on thrombosis and haemostasis (ISTH) score (platelet count, prothrombin ratio, values of fibrinogen and D-dimer levels) were performed regularly. Additional, complementary tests (soluble complexes test, euglobulin lysis test, antithrombin level dosing, activated protein C and factor V dosing) were also performed. ISTH score enables early diagnosis of DIC.

## Introduction

La coagulation intravasculaire disséminée (CIVD) est un syndrome de défibrination grave qui accompagne des situations cliniques bien définies et reflète la défaillance du système microvasculaire dans sa capacité à maintenir la fluidité du sang. La CIVD s´accompagne d´une activation de la coagulation et de la fibrinolyse. Le diagnostic repose sur un ensemble évocateur d´anomalies biologiques [1]. Ces tests ont soit une mauvaise sensibilité soit une mauvaise spécificité pour le diagnostic de CIVD. Différents systèmes de score standardisés ont été proposés, le score de la Société Internationale sur la Thrombose et l´Hémostase (ISTH) est le plus validé, le rendant de ce fait un score de référence. Ce score prend en considération le tableau clinique et une combinaison des tests les plus souvent utilisés en routine [2].

## Méthodes

Nous rapportons trois observations cliniques de CIVD d´étiologies différentes. L´expression clinique de la CIVD est variable selon le contexte pour chaque patient. Les différents paramètres d´hémostase ont été régulièrement réalisés et le système de score ISTH a été quotidiennement calculé (Annexe 1). Ce score a permis de confirmer le diagnostic de CIVD, prédire le risque d´évolution péjorative pour chaque patient, permettant ainsi une prise en charge thérapeutique précoce.

## Résultats

**Cas clinique 1:** il s´agit d´un patient âgé de 89 ans, admis au service d´urologie pour prise en charge d´un adénocarcinome de la prostate (ADK) avec métastase osseuse. Le patient avait présenté une altération de son état respiratoire et neurologique nécessitant son transfert aux soins intensifs. Les explorations cliniques et para cliniques ont conclu au diagnostic d´une pneumopathie et d´une infection urinaire à *Pseudomonas aeroginosa* associé à *Escherichia coli* productrice d´une β lactamase à spectre élargi. Une antibiothérapie a été instaurée. Le score ISTH a été calculé vu l´état de choc septique avec défaillance multi viscérale et l´ADK prostatique métastatique. Ce score était indicatif au premier jour d´hospitalisation d´une possible CIVD compensée. Les examens d´hémostase ont été recontrôlés quotidiennement (Tableau 1). A partir du quatrième jour d´hospitalisation, le patient présentait un score ISTH à5 confirmant ainsi le diagnostic d´une CIVD décompensée. Le dosage de l´antithrombine et de la protéine C activée était respectivement de 30% et de 24%. Le patient était transfusé avec amélioration partielle des troubles de l´hémostase, suivie d´une rechute rapide. L´évolution était défavorable avec installation d´une défaillance multi viscérale, puis décès.

**Tableau 1:** évolution des paramètres hématologiques et calcul du score ISTH pour le patient de l´observation n°1

	J0	J1	J2	J3	J4	J5	J6	J7	J8	J9
**Plaquettes (?10^9^/L)**	75	65	60	49	35	68	18	37	30	18
**Taux de Prothrombine (%)**	68	66	60	40	38	50	33	39	35	30
**D-dimères (ng/mL)**	2494	2250	1940	1840	1522	1510	1463	1332	1458	1966
**Fibrinémie (g/L)**	6,5	6,8	7,5	6,9	5,8	5,4	5,56	5,62	5,89	4,80
**Score**	4	4	4	5	6	4	6	6	6	6

↑hématurie ↑5PFC+2CGR+10 CPS ↑défaillance

J: jour d'hospitalisation, PFC: plasma frais congelé, CGR: concentrés de globules rouges, CPS: concentré plaquettaire standard

**Cas clinique 2:** il s´agit d´une patiente âgée de 52 ans, admise au service de réanimation pour prise en charge d´un état de choc septique avec défaillance multiviscérale en rapport avec une bactériémie à *Escherichia Coli* compliquant une infection urinaire. La patiente était mise sous antibiothérapie. Le calcul du score de l´ISTH (6) a confirmé une CIVD patente dès l´admission. De ce fait, le score ISTH était quotidiennement calculé (Tableau 2). La recherche de complexes solubles était positive confortant le diagnostic de CIVD décompensée avec altération du système de fibrinolyse. Les taux d´antithrombine et de protéine C étaient respectivement à 25% et à 22%. La patiente continuait à altérer son bilan d´hémostase nécessitant le recours à des transfusions avec une amélioration partielle des troubles de l´hémostase suivie d´une rechute rapide. La patiente a fait un infarctus du myocarde avec une évolution fatale.

**Tableau 2:** évolution des paramètres hématologiques et calcul du score ISTH pour la patiente de l´observation n°2

	J0	J1	J2	J3	J4	J5	J6	J7	J8
**Plaquettes (?10^9^/L)**	18	50	32	58	109	36	39	42	52
**Taux de Prothrombine (%)**	41	38	32	45	43	36	38	42	48
**D-dimères ?(ng/mL)**	6542	9591	>10000	7938	>10000	9200	>10000	>10000	>10000
**Fibrinémie (g/L)**	4,20	3,06	2,60	2,51	2,16	2,09	2,05	2,01	2,34
**Score**	6	5	6	4	3	6	6	5	4

↑5 PFC ↑10 CPS + 2CGR

J: jour d´hospitalisation, PFC: plasma frais congelé, CGR: concentrés de globules rouges, CPS: concentré plaquettaire standard

**Cas clinique 3:** il s´agit d´une patiente âgée de 31 ans admise aux soins intensifs pour complément de prise en charge d´une hémorragie de post partum (HPP) compliquant un hématome rétro placentaire (HRP) post-traumatisme abdominal. Une césarienne en urgence avait été réalisée devant une souffrance fœtale. Le bilan biologique préopératoire montrait un bilan hépatique normal et une thrombopénie au bilan d´hémostase. Le bilan d´hémostase refait en peropératoire montrait une chute du taux d´hémoglobine à 8.1g/dL. Une thrombopénie à 48×10^9^/L, un TP à 63%, une fibrinémie à 2.9g/L et des D-dimères à 10000ng/mL. La patiente était en état de choc hémorragique avec un saignement en nappe. Le score ISTH calculé était à 6, ce qui confirmait le diagnostic de CIVD décompensée. La patiente était alors transfusée et une triple ligature vasculaire ainsi qu´un capitonnage étaient réalisés. En postopératoire, le bilan d´hémostase était régulièrement suivi et le score ISTH quotidiennement calculé. Les résultats obtenus sont résumés dans le Tableau 3. Tous les paramètres du score ISTH étaient rapidement corrigés sauf les D-dimères (correction partielle et plus tardive). Le temps de lyse des euglobulines était légèrement raccourci et la recherche des complexes solubles au cours de la CIVD était négative. Le taux du facteur V était à 55%. Les taux d´antithrombine et de protéine C étaient respectivement de 65% et 68%. Devant la stabilité des constantes vitales et du bilan biologique, la patiente était transférée au service de gynécologie.

**Tableau 3:** évolution des paramètres hématologiques et calcul du score ISTH de la patiente de l´observation n°3

	J0			J1	J2	J3	J4
**Plaquettes (x10^9^/L)**	Préopératoire 90	Per opératoire 48	Post opératoire 102	75	100	85	140
**Taux de prothrombine (%)**	100	63	79	75	85	89	100
**D-dimères (ng/mL)**	4270	>10000	>10000	8230	5480	3300	1872
**Fibrinémie (g/L)**	5,11	2,9	3,8	4,2	4,5	4,6	2,8
Score	4	6	3	4	3	3	2

↑6 PFC+3 CGR+10 CPS Transfert au service de gynécologie

J: jour d'hospitalisation, PFC: plasma frais congelé, CGR: concentrés de globules rouges, CPS: concentré plaquettaire standard

## Discussion

L´ISTH a proposé un système de score basé sur des tests d´hémostase de routine; plaquettes, temps de Quick, fibrinogène et D-dimères. Le comité d´experts chargé d´établir ce score a émis des critères diagnostiques pour deux entités la CIVD «compensée» et la CIVD «décompensée» [3-6]. Les critères établissant le diagnostic de CIVD «compensée» devaient être le plus sensible possible alors que le diagnostic de CIVD «décompensée» devait être le plus spécifique [3, 4]. Un total de point supérieur ou égal à 5 étant compatible avec une CIVD décompensée ou clinique, et un score inférieur à 5 évoque sans affirmer une CIVD compensée. Le diagnostic de la CIVD «compensée» ou biologique est retenu pour un total de point supérieur au égal à 7 [7]. Cet algorithme ne peut être appliqué que pour les patients présentant l´une des pathologies associées au développement d´une CIVD [4]. Il faut insister sur la nécessité de répéter les tests régulièrement.

Notre première observation témoigne de la situation où une coagulopathie vient compliquer l'évolution d'un adénocarcinome prostatique en cours du traitement. Cette tumeur est associée parfois à des anomalies de l'hémostase et dans la majorité des cas les patients sont dans un état de CIVD compensée chronique. Toutefois, cet état peut à tout moment basculer vers une CIVD décompensée avec soit des manifestations hémorragiques catastrophiques soit des accidents thrombo-emboliques cliniquement manifestes [8-13]. Le calcul de score ISTH avait permis de classer le patient parmi la population à haut risque de CIVD au stade compensé. Cependant le bilan biologique réalisé durant les trois premiers jours d´hospitalisation n´était pas du tout concluant ni évoquant la suspicion d´une CIVD. Une fois le diagnostic de CIVD décompensée est posé, le score ISTH devenait un score pronostic. Chez notre patient le calcul du score évoquait une évolution défavorable, confirmée par les taux diminués d´antithrombine et de la protéine C.

Dans la deuxième observation, la patiente présentait des troubles de l´hémostase représentés par la thrombopénie qui constitue un marqueur simple de l´existence d´un dysfonctionnement de la balance hémostatique au cours des états septiques grave. Cet état septique aurait entrainé une distribution anormale du sang dans la microcirculation. En effet, la toxine active de l´*Escherichia coli* aurait entraîné à la fois une activation de la coagulation et de la fibrinolyse réactionnelle. La coagulation activée a été exprimée par les taux diminués de prothrombine qui variaient entre 32% et 48% révélant une génération non contrôlée de la thrombine. Les taux de fibrinogène et de D-dimères élevés, constituent les signes de fibrinolyse secondaire active. La dégradation de fibrine en D-dimères a été clairement exprimée par leurs cinétiques respectivement descendante et ascendante. Au cours d´un choc septique, la CIVD évolue assez rapidement [14-17]. La consommation des plaquettes et des facteurs de la coagulation était biologiquement évidente, même pour le fibrinogène malgré son taux persistant à des valeurs élevées, expliqué par l´état inflammatoire systémique. Le score ISTH a permis de confirmer le diagnostic de la CIVD et de prévoir son évolution défavorable, confirmée par les taux diminués d´antithrombine et de protéine C. Les tests biologiques être régulièrement effectués. Des études ont montré que le dépistage de la CIVD par le score ISTH chez des patients septiques était associé à une réduction significative de la mortalité [18-20].

Pour la troisième observation, la patiente présentait, dès son admission, des troubles de l´hémostase qui signent la gravité de l´hémorragie en cours. Les D-dimères élevés confirment l´existence d´un processus de fibrinolyse active. En peropératoire, la consommation des plaquettes s´était accentuée, le TP diminué reflète une consommation brutale des facteurs de coagulation en rapport avec une activation accrue de la coagulation. En conséquence, la fibrinolyse réactionnelle s´est activée, révélée par la diminution des taux de fibrinogènene concomitante à l´augmentation des D-dimères [3-5]. La CIVD s´est déclenchée brutalement en peropératoire et il y avait une concordance entre le bilan biologique et le score ISTH, tous deux en faveur du diagnostic de la CIVD. Le score ISTH a permis de suivre l´évolution qui a été favorable.

## Conclusion

Les trois observations cliniques étudiées ont montré que la CIVD peut être soit décompensée, alors très grave avec des défaillances multiviscérales menaçant rapidement le pronostic vital, soit compensée, témoignant d´une coagulopathie à minima révélée uniquement par une thrombopénie ou une anomalie isolée de certains tests biologiques de coagulation réalisés en routine. L´utilisation du système de score de l´ISTH nous a permis de poser le diagnostic précoce de la CIVD, même au stade compensé, permettant ainsi d´identifier les populations à haut risque en milieu de réanimation. Ces patients doivent alors être surveillés de près et bénéficier de procédures spécifiques supplémentaires diagnostiques et thérapeutiques.

### Etat des connaissances sur le sujet

- La Société Internationale sur l´Hémostase et la Thrombose (ISTH) a proposé un algorithme pour déterminer un score de CIVD;
- Ce système utilise les tests simples réalisés par l´ensemble des laboratoires hospitaliers;
- Le système de score ISTH s´est avéré un outil particulièrement performant pour le diagnostic de CIVD, avec une sensibilité de 91% et une spécificité de 97%.

### Contribution de notre étude à la connaissance

- Les soins prodigués aux patients atteints d´une coagulation intravasculaire disséminée (CIVD) sont largement régis par les caractéristiques cliniques et doivent être surveillées de près;
- La prise en charge multidisciplinaire précoce de la CIVD offre des chances de survie accrues aux patients;
- En raison du manque de données probantes guidant le diagnostic et la gestion, cette série ajoutée à la liste des cas publiés, peut éventuellement contribuer aux futures revues systématiques sur le sujet.

## Annexe

**Annexe 1**: système de scores de l´ISTH pour le diagnostic de la CIVD
